# A Literature Review on Whether Immobilization of the Shoulder in External Rotation Improves Healing and Prevents the Recurrence of Acute Shoulder Dislocations

**DOI:** 10.7759/cureus.80713

**Published:** 2025-03-17

**Authors:** Yasmin H Kazim

**Affiliations:** 1 Emergency Department, Rashid Hospital, Dubai Health Authority, Dubai, ARE

**Keywords:** external rotation, immobilization, internal rotation, recurrent shoulder dislocation, shoulder dislocation

## Abstract

Shoulder dislocations are one of the most frequent joint dislocations, with recurrent dislocations being a common complication. Common practice is to immobilize the shoulder in internal rotation for two to three weeks following closed reduction. However, recent literature suggests immobilization in external rotation can be an alternative management strategy. External rotation braces have been manufactured over the years to support and maintain this position.

This literature review aims to gather current evidence on the conservative management of primary traumatic anterior shoulder dislocations and compare the immobilization of the shoulder joint in internal and external rotation.

A literature search and review was performed using PubMed and Google Scholar. Key phrases and words that were used in the search engines included “shoulder immobilization” AND “external rotation”, “anterior shoulder dislocation immobilization” AND “external rotation”, “reduced recurrence rate of shoulder dislocation” AND “external rotation”, “external rotation immobilization” AND “Bankart lesion” and “internal versus external rotation” AND “shoulder dislocation”.

The reviewed articles included were dated from 2014 to 2024 but additional valuable studies dating earlier than 2014 were also included to provide a foundation of understanding to this review.

Included were nine clinical studies and randomized controlled trials, three cadaveric studies, eight studies focusing on magnetic resonance imaging (MRI), magnetic resonance arthrography (MRA) or arthroscopy, and seven systematic reviews and meta-analyses.

Research revealed improved coaptation of the labrum on the glenoid rim in external rotation not only in cadavers but also in patients with the aid of various imaging techniques. However, these findings were not consistently observed when translated into clinical trials.

Based on the available data presented in this literature review, there remains a deficiency in evidence to exclusively support the use of external rotation immobilization over conventional internal rotation immobilization after primary traumatic anterior shoulder dislocations. External rotation immobilization may benefit a specific population, particularly those that fall in the 20-40-year-old age group, with a specific injury pattern, such as Bankart lesions and greater tuberosity fractures; therefore, further studies are required to determine who will benefit the most from such interventions.

## Introduction and background

Shoulder dislocations account for 50% of all major joint dislocations with anterior dislocations making up about 95% of shoulder dislocations [1]. In the United States, the incidence of shoulder dislocations is estimated to be 25.2 per 100,000 person-years, reaching as high as 169 per 100,000 person-years in the young and active population [2,3]. The recurrence of dislocations and instability ranges from approximately 20-67% and can approach 100% in certain age groups [4,5]. The overall five-year incidence rate of recurrent shoulder dislocations is estimated to be 4.2 per 100 person-years [6]. Gender, age at first dislocation, hyperlaxity, greater tuberosity fractures, and participation in contact sports among other factors have been suggested to contribute to the recurrence rate of shoulder dislocations and joint instability [7,8].

Typically, treatment entails a form of analgesia or sedation, a closed reduction, radiographic imaging, bracing equipment, orthopedic consultations, or outpatient appointments. Upon the reduction of a primary anterior shoulder dislocation, the shoulder is immobilized in a sling, with or without a swathe, with the arm held in adduction and internal rotation (Add-IR) for about one to three weeks during which time patients are given time off work to recover or in the case of athletes - taken off the game. This treatment plan demonstrates how shoulder dislocations can financially burden the healthcare system, and perhaps even the athlete, especially if they are recurrent [9,10].

Shoulder dislocations are commonly associated with Hill-Sachs deformities (35-40% of anterior shoulder dislocations), bony Bankart lesions (5% of patients), soft tissue Bankart lesions (90% of patients under 30 years of age with anterior dislocations), and greater tuberosity fractures (10% of patients) [11]. The aim of shoulder immobilization after a dislocation is to allow for joint healing and immobilization remains standard practice after any joint dislocation [12]. Furthermore, immobilization reduces pain and, in turn, increases patient satisfaction. Unfortunately, immobilization, whether for less than a week or for longer than three weeks, fails to show a reduction in recurrence rates, as recurrence rates remain unchanged regardless of the period of immobilization [9].

Shoulder dislocations can negatively impact the quality of life. Those who develop recurrent and chronic shoulder instability have an even greater negative impact as pain persists, leading them to change their sporting activity or job due to impaired performance [13,14]. Even though a large number of athletes may be able to return to play within two to three weeks after a shoulder dislocation, the risk of recurrent instability and pushing the athlete into this vicious cycle remains high [15].

Contrary to immobilizing the shoulder in internal rotation, immobilization in adduction and external rotation (Add-ER) has been proposed to reduce the rate of recurrent shoulder dislocations [16]. This proposal was based on observations whereby it was noted that labral separation and displacement were significantly reduced when the shoulder was immobilized in adduction and external rotation rather than when compared to an adducted internally rotated position [17]. Furthermore, this position increased tension over the subscapularis, allowing the soft tissue to be positioned more favourably over the glenoid [9]. Hatrick et al. used human cadavers to measure the contact force between Bankart lesions and the glenoid in a range of rotations starting with 60° of internal rotation and moving through till 45° of external rotation was reached [18]. They noted that there was zero contact force when the shoulder was in internal rotation and that as the shoulder was shifted into external rotation, the contact force increased to a maximum of 45°. It was hypothesized that the higher the contact force, the higher the rate of healing, but the degree of external rotation that was most accepted by patients was only 10°. This cadaveric study positively supported the findings and recommendations of Itoi et al. [16,17].

In further support, a meta-analysis conducted by Hurley et al. concluded favorable outcomes of immobilization in external rotation after traumatic primary anterior shoulder dislocations in terms of a lower recurrence rate and a higher return-to-play rate when compared to immobilization in internal rotation [19].

Although Itoi et al. demonstrated the reduction of recurrent shoulder dislocations using external rotation as the immobilization of choice - the best outcomes being noted in candidates below the age of 40 - only some meta-analyses supported these findings while others reported the contrary with lack of reproducibility [20,21].

Aim and objectives

This literature review aims to gather the most recent evidence on the management of primary traumatic anterior shoulder dislocations and to conclude if there is enough evidence to support the use of external rotation as a superior method of shoulder immobilization in providing improved recovery and reduced rates of recurrence of shoulder dislocations.

This review will delve into identifying the anatomical differences between internal and external rotation immobilization in relation to the healing shoulder. Additionally, it will look into identifying the degree of external rotation that would bring about the best results and the degree that is clinically tolerated by patients. Other objectives include the identification of the period of immobilization that brings about better healing and the contributory factors and confounders of recurrent shoulder dislocations and shoulder joint instability. Lastly, this review will aim to identify which immobilization method results in a quicker return to activities, reduced recurrence over time, and healing of Bankart lesions.

Method

To obtain and study the most recent available evidence on the topic of matter, PubMed and Google Scholar were used. Various combinations of the key phrases and words that were used in the search engines included: “shoulder immobilization” AND “external rotation”, “anterior shoulder dislocation immobilization” AND “external rotation”, “reduced recurrence rate” AND “shoulder dislocation” AND “external rotation”, “external rotation immobilization” AND “Bankart lesion” and “internal versus external rotation” AND “shoulder dislocation”.

The main focus was on randomized control trials (RCT) and meta-analyses (MA), while other studies looking at the immobilization comparison were reviewed, in addition to determining whether findings were consistent or suggested otherwise. Relevant articles cited within other articles were also reviewed.

With the aim of gathering the most recent evidence available on shoulder immobilization in internal versus external rotation following a primary anterior shoulder dislocation, research from 2014 onward was analyzed. However, with the intention of being able to build a basis and understanding of where the subject lies today, several studies of value dating prior to this, such as the original studies by Itoi et al., which introduced external rotation as a novel technique for immobilizing the shoulder, and several other valuable clinical and imaging studies dating prior to the last decade were also included in this review.

Included were articles focusing on primary traumatic anterior shoulder dislocations, articles studying external rotation immobilization or comparing external rotation to conventional internal rotation immobilization, studies that had a defined follow-up period, studies that mentioned a recurrence rate of shoulder dislocations, and articles that were available in English.

Articles that included recurrent shoulder dislocations or operative management for shoulder stabilization were excluded. Papers that included patients with various shoulder joint or humeral fractures were also excluded. Additionally, discontinued trials, reviews and case reports were excluded from the analysis.

The date of the last search was July 2024. Nine clinical studies and randomized controlled trials, three cadaveric studies, eight studies focusing on magnetic resonance imaging (MRI), magnetic resonance arthrography (MRA) or arthroscopy, and seven systematic review meta-analyses were included.

Of note, no formal data analysis was conducted in this literature review.

## Review

Identifying the anatomical differences between internal and external rotation immobilization in relation to the healing shoulder and how this can impact healing

Shoulder dislocations usually involve variable amounts of damage to the soft tissue surrounding the glenohumeral joint. Lesions may include rotator cuff tears, superior labral anterior-posterior (SLAP) lesions, anterior labral periosteal sleeve avulsions (ALPSA), glenoid defects or greater tuberosity fractures; however, the most common lesion involved is the Bankart lesion with its presence in up to 97% of primary shoulder dislocations [20,22].

Bankart lesions are tears to the anterior-inferior labrum and may include tears at the glenoid rim. The labrum is vital for glenohumeral stability; therefore, an insult to the labrum will increase the susceptibility to recurrent shoulder dislocations and instability [23]. How tissues are positioned or approximated after an acute dislocation, and henceforth during healing, will significantly impact stability. Fifty to eighty percent of all patients with Bankart lesions will achieve healing, thereby reducing the rate of recurrence, but the question lies in what the best position would be to bring out such healing. If the space between the glenoid and capsulolabral complex can be better approximated to allow for adequate tissue apposition, the rate of recurrence after a primary anterior shoulder dislocation is likely to be lower [23,24].

The anterior-inferior structures of the shoulder that provide stabilization are relaxed when the shoulder is adducted and internally rotated and therefore allow medial displacement of the detached glenoid labrum [24]. Using cadaveric studies to introduce a new hypothesis, Itoi et al. were able to produce simulated Bankart lesions and demonstrate a graduation of coapted edges as the adducted shoulders moved through a range from full internal rotation to 30 degrees of external rotation, thereby introducing a new method of immobilizing primary traumatic anterior shoulder dislocations. Moreover, as gradual abduction was introduced, coaptation became less regardless of the degree of external rotation [25]. Another cadaveric study was performed whereby the contact force between the glenoid labrum and the glenoid was measured in three positions: 60° of internal rotation, neutral rotation, and 45° of external rotation. It was noted that external rotation significantly increased the contact force between these structures, proposing better Bankart healing [24].

With the aid of MRI, the coaptation of Bankart lesions in patients with anterior shoulder dislocations was further investigated; first analyzing the coaptation in internal rotation, with a mean of 29°, and then in external rotation, with a mean of 35° [17]. Separation and displacement of the anterior-inferior portion of the labrum from the glenoid rim, along with the detached area and the opening angle of the anterior-inferior portion of the capsule, were greatly reduced when the shoulder was in external rotation than when it was in internal rotation. Furthermore, the detached length was significantly shorter with the shoulder in external rotation.

In the study by Liavaag et al., computer tomography (CT) and MRI were used to evaluate Bankart lesions while MRA was performed after the period of immobilization. Similar findings were discovered in terms of reduced labral separation in the externally rotated group, as well as a reduction of Bankart lesions as a whole; however, there was no difference noted in terms of labral displacement and the detached area between the internally and externally rotated groups [26].

Through arthroscopy, Hart and Kelly were able to get to a similar conclusion as Itoi and colleagues, whereby external rotation did, in fact, tighten the anterior-inferior portion of the capsulolabral complex. They described better reduction when 30 degrees of abduction was coupled with 60 degrees of external rotation [27].

Siegler et al. examined 23 shoulders using MRI assistance. In all 23 shoulders, hemarthrosis was almost always systematically present [28]. Itoi et al. described that the pressure induced by hemarthrosis was one factor that resulted in the maintenance of anterior capsular detachment following a shoulder dislocation. With external rotation, it was noted that the distribution of the hemarthrosis was altered to some degree in 75% of the cases while anterior joint effusion was reduced in all cases and completely reduced in 13% of the cases. External rotation produced a variation of labral coaptation while 21% of the cases were fully coapted in external rotation. These findings supported external rotation as an effective method in reducing the labral separation to some degree [17].

Complementary to this, Itoi et al. undertook another study consisting of 31 patients. After initial reduction, an MRI was taken of each shoulder in four different positions: adduction and internal rotation (Add-IR), adduction and external rotation (Add-ER), 30° of abduction and 30° of external rotation (30Abd-30ER), and 30° of abduction and 60° of external rotation (30Abd-60ER). Again, the separation, the displacement of the labrum, and the opening angle of the capsule were measured. When compared to Add-IR, Add-ER significantly reduced the anterior and anterior-inferior labral separation but not the inferior labrum separation; and although 30Abd-30ER had a slight impact on reducing the anterior-inferior and inferior labra, the most significant improvements were noted in the 30Abd-60ER even when compared to external rotation alone. The same can be said regarding the displacement and opening angle of the joint capsule, concluding that 30Abd-60ER was the best position to aid in the effective reduction of a Bankart lesion. In part, these results favored external rotation for an improvement in labral coaptation after primary traumatic shoulder dislocation [29]. Momenzadeh et al. assessed and analyzed the displacement, separation, and opening angle parameters in 20 patients distributed across the two groups, internal rotation (IR) and external rotation (ER), and were able to obtain similar favorable results. All variables showed a reduction in the ER group indicating labral coaptation to a near anatomical position when the arm was immobilized in an external rotation position [30].

In an MRI-controlled prospective study by Königshausen et al., using 15° to 30° external rotation for three weeks along with a five-year follow-up, they were able to show positive results with a reduction of recurrence and instability rates. The MRI studies performed after the initial trauma showed significant improvement in the position of the labrum relative to the glenoid rim with external rotation in comparison to internal rotation. When re-imaged six weeks later during a follow-up, the labral position showed a significant improvement; and when positioned in internal rotation for comparison, there was no significant difference with respect to the dislocation thereby indicating good healing [31].

With regards to the rate of healing, there is no reported difference in the healing rate of lesions initially identified between the IR and ER groups [12].

Identifying the degree of external rotation that brings about the best results and the degree that is clinically tolerable by patients

Through previous cadaveric studies, we understand that the contact force between the structures involved in a Bankart lesion improves through the range of external rotation of the shoulder, with a maximum contact force noted at 45° of external rotation [24]. Some studies even favor the coupling of abduction and external rotation.

Itoi et al. produced positive results in their trial at 10° of external rotation, stating that 30° of external rotation was intolerable by patients [20]. Using 0-5° of external rotation, Whelan et al. failed to show favoring results but attributed this possibly to the small sample size (60 patients) and moderate loss to patient follow-up (17%) [32]. Their attempt to immobilize patients’ shoulders in anything more than 5° of external rotation was poorly tolerated and compliance to wearing the brace was only 75% of the total immobilization period. Contrary to this, in the trial by Murray et al., 15° of external rotation was just as tolerable as internal rotation, however, the results only significantly favored external rotation in the 20-40-year-old age group when sub-analyzed [12]. 

In the study by Heidari et al., 102 shoulders were equally randomized into two groups, 51 of which were stabilized in a position of 15° of abduction and 10° of external rotation. The Add-IR group was found to have a 33.3% recurrence rate while the abducted externally rotated (Abd-ER) group had only a 3.9% recurrence rate; this was statistically significant, particularly for the 31-40 age subgroup analysis. When compliant, the Abd-ER method was also associated with absolute and relative risk reductions in comparison to Add-IR. Unfortunately, regardless of the better outcome in the Abd-ER group, the rate of noncompliance was higher in the Abd-ER group; the more a shoulder is placed in external rotation, the more uncomfortable it becomes for the patient, therefore necessitating the discovery of the degree that not only brings out sufficient healing but also sufficient patient comfort. The noncompliance in this study was attributed to the newly designed brace to help maintain the shoulder in an Abd-ER position. Furthermore, one of the limitations of this study was the lack of investigation and documentation of the ability to maintain the glenohumeral joint in external rotation using the new brace design [33].

There have yet to be larger studies with consistent results comparing different degrees and variations with the addition of abduction and a combination that is closest to neutral yet still produces sufficient contact force to improve patient compliance and comfort.

In a step toward this, a small study was carried out by Sullivan et al. to simulate conditions a patient would have to go through using a commercial brace to help maintain external rotation. Observations were made in several aspects. Significant differences were noted between commercially available braces in their ability to achieve the degree of rotation it is advertised to produce and in maintaining shoulder external rotation thereafter. It was noted that during activities of daily living, the degree the brace was initially set at altered, and only some can be reset to a close-enough degree when re-applied by the patient. Additionally, there was a discrepancy among comfort ratings and cost between the various braces [34]. Hatta et al. elaborated on this by looking at four distinct braces - two ER braces and two Abd-ER braces [35]. They reported that the various braces in their study were able to maintain the initial shoulder position even after daily activities and re-application but noted that the Abd-ER braces were less comfortable during daily activities when compared to the ER braces. These four braces were similarly rated in terms of user-friendliness. Looking further into comfort as an important factor involved in clinical compliance, Hatta et al. noted that abduction and external rotation of the shoulder can be acceptable, although, with an abduction of 30°, a larger degree of external rotation is associated with greater discomfort when compared to narrower angles and adduction [36]. These specific studies were all performed on healthy individuals who wore the selected braces for a short period of time, ranging from 30 minutes to 24 hours. Abduction and external rotation have favored Bankart reduction according to the studies described above, but as this might not be as comfortable as standard immobilization, larger clinical trials have yet to be done to determine a clinically acceptable immobilization position [29].

Identifying the number of weeks of immobilization that brings about better healing

Soft tissue healing in the form of granulation tissue typically occurs by the seventh to tenth-day post-injury and increases its tensile strength by three weeks. The formation of granulation tissue fills the gaps and unites soft tissue. For a very long time, immobilization of the shoulder after closed reduction has been the standard treatment, aiming to promote more effective healing. In the search to determine the appropriate length of immobilization, most studies used and recommended three weeks of immobilization [16,21]. Opposing this, some researchers have found no difference in the recurrence of shoulder dislocations when comparing one week and three weeks of immobilization in the conventional internal rotation position [37]. Zhang et al. reported that immobilization in the ER for three weeks could significantly reduce the recurrence rate (P=0.001) but found no difference when immobilized for four weeks [38]. In agreement, Hurley et al. and Schliemann et al. concluded that immobilization for longer than three weeks was not shown to be associated with better outcomes [19,39]. Various other studies have also reported a lack of difference in terms of the healing Bankart lesion between an immobilization period of three weeks versus five weeks [21].

It must be kept in mind that immobilization for a given number of weeks must also take into account the number of hours per day the patient remains compliant with its use. Certain studies considered a patient to be compliant if the brace was worn for longer than 16 hours, while others considered a patient compliant “full-time” if the brace was only removed for changing clothes or taking a shower. Focusing on the studies where the patients wore the brace full-time, the outcome shows a significant difference in an event occurring (shoulder dislocation) when comparing external rotation and internal rotation groups; indicating that immobilizing the shoulder full-time during the period of healing is crucial and that the immobilization of the shoulder in the external rotation position is favored [21]. 

Hatta et al. compared and contrasted four different shoulder braces delving into patient comfort and compliance. These braces were to immobilize healthy shoulders in Add-IR, Add-ER, 30Abd-30ER, and 30Abd-60ER for 24 hours. After completing the period of immobilization, the participants were asked to rate the level of discomfort of bracing for overall and individual activities. The brace that provided immobilization at 30Abd-60ER was rated the most uncomfortable and it is presumed such braces will prove to have poor compliance [35]. While certain studies surprisingly reported no increase in discomfort with the shoulder immobilized in external rotation, there were some reports of discomfort and inconvenience of positioning and hence compliance with immobilization in external rotation may continue to be a concern. Furthermore, several RCTs have indicated an equal or even higher rate of compliance to external rotation braces, which could only be explained by the possibility that its benefits were thoroughly explained to the patient with regards to the decreased risk of recurrence of shoulder dislocations, and such explanation remains of utmost importance to be able to get patients to adequately maintain the required ER position [39]. 

Identifying contributory factors to the recurrence of shoulder dislocations and joint instability

Several factors have been identified to affect the recurrence of shoulder dislocations, such as age, age at first dislocation, male gender, and associated patterns of injury such as the presence of a Hill-Sachs lesion or the like, poor compliance or limited usage of the shoulder brace, and contact or overhead sports participation [12].

A bimodal distribution has been described in men and a unimodal distribution in women. Men had a higher incidence between the ages of 15 and 44 and above the age of 85, peaking at 20 to 29 years while women peaked between the ages of 55 and 74 [40,41]. In the 25-year epidemiological study by Becker et al., they noted the highest number of shoulder dislocations among women occurred between the ages of 80 and 89 [3]. Although Vavken et al. were able to see a difference after age stratification, suggesting age to be a confounder, the amplitude of this difference was quite small indicating the possibility of additional covariates [23].

The age of onset of primary shoulder dislocation is reported to be the most significant risk factor for recurrence. Several studies have found the younger population to be at a greater risk for developing two or more subsequent recurrent dislocations. Patients who are below the age of 20 at the time of the initial injury have a higher risk of recurrence when compared to those aged 30-40 [12]. Patients younger than 30 years of age are generally more likely to sustain a recurrent dislocation than patients older than 30 [37].

Male gender was associated with a higher risk of recurrence especially in the 10-19 and 20-29 age groups [6,41].

Immobilizing the shoulder on the same day of the dislocation, as opposed to immobilization on day two or three, has been shown to have better outcomes in the ER group than the IR group [20]. 

Positioning of the shoulder can affect the tissue tension but an additional implicated factor for recurrence is the presence of an intra-articular hematoma after an acute dislocation. This is because the intra-articular hematoma can cause displacement of the capsulolabral complex from its anatomic origin. The external rotation position helps increase the subscapularis tension, creating an intra-articular tamponade effect that allows for a reduction of the hematoma formation and further improves the approximation of the Bankart to the glenoid. Hematoma evacuation by means of arthroscopic lavage can also aid in the reduction of recurrent dislocation [19,24].

There is no doubt the pattern of tissue damage, such as bony or ligamentous Bankart lesions, the associated anatomic parameters, such as the integrity and function of the subscapularis, as well as the distension of the capsule and the glenohumeral ligaments, all play vital roles in the healing and ultimately the recurrence of a shoulder dislocation [2,23]. Opposingly, Wasserstein et al. did not find a significant difference in terms of recurrent instability or dislocations when looking at bony Bankart lesions or Hill-Sachs lesions as contributory factors [42].

According to Murray et al., unhealed labrums, regardless of the immobilization position of choice, are a clear cause of the recurrence of shoulder dislocations (P=0.0001). Using their MRA findings, they noted no difference in the healing rate between ER and IR groups with identified lesions but they did, however, note a higher recurrence rate with a healed labrum in the IR group, although this was not statistically significant (P=0.47) [12].

Among the 20- to 40-year-old subgroup, immobilization in the ER particularly reduced the rate of recurrence but there was no difference between the ER and IR groups for the subgroup younger than 20 years of age [12,21,43].

Generally, it appeared that patients in the external rotation group did better in terms of recurrence when comparing several subcategories such as gender, contact or overhead sport, or competitive sport, but this lacked statistical significance [12]. 

A 25-year follow-up study conducted by Hovelius and Rahme found that 50% of primary shoulder dislocations were related to some variation of sports activities - contact sports in particular, but surprisingly, sports did not impact the rate of recurrence. Additionally, both gender and minor bony Bankart lesions did not affect the recurrence of shoulder dislocations. Their 25-year follow-up allowed them to reveal that with time, arthropathy worsened. Patients who were 25 years of age or younger at the time of the primary shoulder dislocation had less arthropathy after the 25-year period than patients who were aged 26-40 years at the time of dislocation (P=0.01). Furthermore, the 12-22-year-old age group had a higher recurrence rate than those above 22 years of age. After the 25-year period, it appeared that 29% of patients who had two or more recurrent dislocations had stabilized with time. Alcoholism was demonstrated to be an additional risk factor for increased arthropathy [43].

Co-morbidities such as diabetes mellitus, hyperlipidemia, and hypertension were not shown to affect the rate of recurrent dislocations [6]. Nevertheless, medical conditions involving generalized ligamentous laxity or hypermobility, such as Ehlers-Danlos Syndrome, are known to be risk factors for shoulder dislocations and chronic instabilities [9]. In addition, epilepsy also contributes to being a risk factor for recurrent shoulder dislocations (4.8%) [10].

Which immobilization method results in quicker return to activities, reduced recurrence over a period of time, and healing of Bankart lesions

It has been well-reported that the recurrence rate of shoulder dislocations and instability after traumatic primary anterior shoulder dislocations using nonoperative management is extremely high, especially in young patients. The reasoning is suggestive of the inadequate healing of the tissue-bone interface of the Bankart lesion when the arm is immobilized in internal rotation [12,24]. As seen using MRI assistance, immobilization in external rotation anatomically reduces the separation and displacement of the anterior-inferior portion of the labrum, which internal rotation fails to do [17]. Not only has external rotation immobilization shown to have better outcomes, but these outcomes are significantly better among 20-40-year-old patients with primary anterior shoulder dislocations [12,19,21].

According to Patrick et al., attempting to conceal allocations to IR or ER groups is impractical and only a single-blinded allocation could ideally be possible. Furthermore, they suggest the variation in yielded results among recent RCTs could be attributed to the differences within the studied populations, leading to the possibility of identifying and choosing specific patient groups that could respond favorably to immobilization in external rotation [2]. Hatta et al. suggest that the choice of brace used in each study may also affect the variable outcomes, as their ability to maintain the desired position of immobilization will impact the quality of treatment provided [35].

Several earlier RCTs, systematic reviews and meta-analyses (SRMA) looking at the outcomes of immobilization in external rotation when compared to internal rotation have failed to find a significant difference in any clinical parameter between the protocols. As seen with Whelan et al., the results of their RCT indicated immobilization in external rotation did not produce a significant benefit when compared to sling immobilization in internal rotation in the prevention of recurrent dislocations after primary anterior shoulder dislocation; however, only a 0-5° angle of external rotation was used during their immobilization, and while patients were followed up for one year, they had only worn their braces for about 75% of the required immobilization period [32].

During the pooling of data for the meta-analysis by Liu et al., they found no significant difference in recurrence rate among the various age groups, nor did they find any significant difference between the ER and IR groups. They delve into the reasoning behind this and noted that the trials included in their meta-analysis did not exclude patients with probable Hill-Sachs lesions and rotator cuff tears, both of which are known to cause instability of the shoulder joint without appropriate treatment [44]. Similarly, in 2016, a meta-analysis of RCTs by Whelan et al. was conducted, which included only six randomized or quasi-randomized controlled trial studies, all with relatively short follow-up periods. Their conclusion suggested that immobilization in the ER was not significantly more effective than immobilization in IR [45].

Since the publications of newer RCTs, more recently updated meta-analyses have been able to further analyze these differences. The pooled data from the meta-analysis by Hurley et al. indicated that external rotation immobilization did, in fact, result in a lower rate of recurrent dislocation after primary anterior shoulder dislocation, including the younger patients who may be at higher risk of recurrent instability. Although there were concerns that immobilization in external rotation would be highly uncomfortable for patients, surprisingly, patient compliance was reported to be higher with this particular protocol when compared and contrasted [19]. Furthermore, the patients who were treated with external rotation were found to have a higher rate of return-to-play at the same pre-injury level and this was supported earlier by Heidari et al., whereby they noted the group of patients who were immobilized in an ER position had a higher rate of returning to pre-injury sportive activities by 24 months than those who were treated by IR immobilization (P=<0.001) [19,33].

In an 18-year follow-up by Itoi et al., they observed no significant difference in the recurrence rate or rate of instability between the ER and IR groups (P=0.100). They also reported no difference in return to sports. A significantly higher rate of surgical intervention was noted in the IR group (P=0.038), but for the patients who survived the follow-up period, there were no significant differences between the groups in terms of clinical outcome. The lack of significant findings was related to the poor follow-up rate of only 28% [46].

Discussion

At a glance, shoulder dislocations appear to be a simple matter. But, in fact, the complexity of it can be overwhelming, perhaps even intriguing. The shoulder's ball-and-socket structure allows for a large range of motion and, therefore, frequently predisposes the joint to dislocations and instabilities. While it comprises half of all major joint dislocations, a gross reduction process is far from sufficient in terms of management. Over decades, researchers have tried, and failed, to reduce the high rate of recurrent shoulder dislocations. And while many factors and confounders have been studied, the ideal management has yet to be standardized.

In 1999, Itoi et al. introduced a novel concept of immobilizing the shoulder in an external rotation, rather than the conventional internal rotation [25]. This concept arose with the help of their research on cadavers with simulated Bankart lesions. This study revealed better ‘coaptation’ of the structures involved when the shoulders were placed in external rotation, particularly that of the Bankart lesion. Coaptation improved further with the coupling of shoulder abduction. With the aid of MRI, Itoi et al. were able to identify the effects of external rotation on the reduction of the detached area and the opening angle of the anterior-inferior portion of the shoulder joint capsule. In addition, the length of detachment was significantly shorter [17]. Scientifically, when anatomical structures are separated and disrupted during an injury, the distance and alignment between the involved structures during healing is what affects outcomes. Murray et al. report that a healed Bankart lesion can reduce the recurrence of shoulder dislocations, and with external rotation, the involved structures of the shoulder are anatomically reduced and therefore optimal healing is expected [12].

Cadaveric and MRI studies that followed later had continued to show that shoulder joints placed in external rotation, with or without abduction, could better approximate the glenoid to the glenoid labrum producing a contact force that increased as the shoulder was placed further into a greater degree of external rotation [18,24,29]. Itoi et al. demonstrated optimum findings with 30 degrees of abduction and 60 degrees of external rotation rather than with external rotation alone [29]. This notion of ideal reduction, with a reduction in labral separation and displacement, is thought to lower recurrence rates of shoulder dislocations. As researchers went from cadaveric studies to clinical trials, there initially appeared to be positive results with the immobilization of shoulders in external rotation; however, with time, they have failed to replicate results consistently.

There have been numerous suggestions of what could possibly contribute to these discrepancies. The degree of healing will depend on what injury is to be healed. While the majority of shoulder dislocations are known to be associated with Bankart lesions, Liavaag et al., Königshausen et al., and Momenzadeh et al., among others, had restricted their study population to patients with primary shoulder dislocations alone, excluding other associated osseous and ligamentous lesions [30,31,47]. On the other hand, clinical studies by Itoi et al., Hart and Kelly, Liavaag et al., Siegler et al., and Whelan et al. included patients with a range of lesions in their study sample such as shoulder subluxations and instability, Hill-Sach lesions, labral and rotator cuff tears, greater tuberosity fractures and hemarthrosis [16,26-28,32]. The extent of the underlying injury can influence the magnitude of benefit that is expected to be seen with external rotation immobilization.

In medical practice, there are several methods of closed shoulder reduction, such as the Spaso method, the Hippocratic method, the Kocher method, and the Stimson method, to name a few. Certain methods, such as the Hippocratic method, are outdated and not recommended due to complications such as brachial plexus injury and axillary vessel injury. Similarly, the Kocher method carries its own complications and has been associated with injuries such as humeral shaft fractures, axillary vein rupture and rotator cuff injuries [48]. In the RCT by Itoi et al., several methods were used to reduce the shoulders of patients included in the trial, some methods of which are known to have high complication rates [20]. Other trials and cohorts included in this review did not mention the methods of reduction used. It may be possible that the chosen method of closed reduction could, if associated with a complication, influence healing and rates of recurrent shoulder dislocations.

Cadaveric and MRI studies had the upper hand in testing greater degrees of external rotation to achieve favorable results. Miller et al. produced evidence that 45 degrees of external rotation produced the highest contact force between the labrum and the glenoid [24]. Furthermore, the controlled MRI laboratory study by Itoi et al. demonstrated optimum findings with 30 degrees of abduction coupled with 60 degrees of external rotation, which was more significant when compared to external rotation alone [29]. When translating this view to clinical subjects, researchers encountered difficulty. The expression of this was observed in a study by Itoi et al., whereby within their pilot study, they documented an attempt to immobilize the shoulders of patients in 30 degrees of external rotation [16]. As this was poorly tolerated, the external rotation angle was decreased to 10 degrees. Murray et al. also mentioned a preliminary trial prior to their RCT to investigate an acceptable degree of external rotation. They reported patients were only able to tolerate 15 degrees of external rotation and, hence, this was the degree used to carry out their trial [12]. Clinical studies have since then used a degree of external rotation ranging from 0° to 15° at most, with a few trials reaching close to 30°. Heidari et al. coupled 15° of external rotation with a 10° degree of abduction. They reported a recurrence rate of 33.3% in the Add-IR group and 2.4% in the Abd-ER group (P=<0.001) among those who were compliant to brace use [33]. It is possible that this variability in the angle of immobilization additionally contributes to the differences in results seen between various clinical trials.

Within these studies, different external-rotation braces were used, such as the Don Joy Ultrasling ER, 15° version and the Don Joy Ultra Sling II ER (DJO Global, Carlsbad, California, US). While commercially available external rotation braces exist, the cost remains high and therefore inconvenient for some to purchase. In some studies, such as those by Itoi and colleagues, and by Momenzadeh et al., an external rotation brace was compiled using cast materials, wires, and a sponge. Heidari at el. produced a stabilizing brace using hard polyethylene, Velcro, and an adjustable metal bar to establish their 15°Abd-10°ER angle [30,33]. And while three to four weeks were standardized throughout cohorts and trials, the ability for any of these braces to maintain the desired angle during activities of daily living and after its reapplication by the user remained questionable. The lack of ability to maintain the angle of study can, no doubt, contribute to the variation in reported results.

The trial by Heidari et al. was the first randomized control trial to compare Abd-ER and Add-IR in patients with primary anterior shoulder dislocations [33]. With the introduction of abduction clinically, a significant difference was seen in patient cooperation between Abd-ER and Add-IR groups. There was a higher rate of noncompliance in the Abd-ER group, with up to 19.6% noncompliance when compared to the Add-IR group, which had only a 5.8% noncompliance rate (P=0.03). Regardless of the type of ER brace used across trials, although external rotation immobilization is frequently regarded as being uncomfortable, pooled data analysis indicated the contrary; the compliance rate with ER braces was deemed overall similar to the compliance rate of IR braces.

Previous MRI studies have proven external rotation immobilization for three weeks to be effective in reducing the detached labrum to the glenoid [16,49]. Currently, no supporting evidence indicates that immobilizing the shoulder for longer than three weeks could provide any additional benefit to the healing shoulder [19,39]. However, just as compliance to the type of brace is important, the number of hours per day over the course of these three weeks is just as important. Educating individuals on the benefits of the treatment may play a vital role in compliance.

Another explanation for conflicting findings is the use of heterogeneous populations. An example of this is the study carried out by Finestone et al., which was exclusively done on a small population of young military men [50]. Differences among ethnic groups have been reported by Becker et al., with White and Caucasian groups accounting for the largest number of all shoulder dislocations [3]. Age in itself appears to be a complex non-modifiable covariate. Itoi et al. used a wide age range of patients [12]. Later studies began reducing this age range to a more focused range. Itoi et al., Liavaag et al., and Murray et al., documented a high recurrence rate between the ages of 16 and 30. They report that patients below the age of 20 to 30 at the time of the initial shoulder injury tended to have a higher recurrence rate when compared to patients between the age of 30 and 40 [12,16,47]. But just as an age younger than 40 represents an independent risk factor for the recurrence of shoulder dislocations, favorable results with external rotation immobilization have been reported by Itoi et al., Taskoparan et al., and Murray et al. in the 20-40 age subgroup [12,20,51]. These findings indicate the significance of the role age plays in cases of shoulder dislocation, and age subgroup analysis encourages researchers to try to understand where these result variations come from.

The bimodal nature seen in males between the ages 15 and 44 and above the age of 85 can be attributed to the participation in sporting activities of the younger age group and traumatic accidents, such as falls, in the older age group [3,41]. Hovelius et al. report a high recurrence rate of 72% in the 12-22-year-old group [52]. This percentage was lower in the 23-29-year-old age group and the above-30-year-old age group with 27% and 38%, respectively. Furthermore, 38% of patients between the ages of 12 and 25 underwent surgical stabilization at some point [9,52]. These results reflect the presumption that younger age groups participate in sporting activities. On the other hand, the unimodal distribution seen in females between the ages of 55 and 74 can be explained by the possible effects of estrogen withdrawal on the tendons in post-menopausal women [41].

Nevertheless, throughout the years, study samples within the various trials have shown to have a larger number of males than females. This could be due to the fact that there is a higher number of males partaking in sporting activities than females. Hovelius and Rahme attribute 50% of primary shoulder dislocations to be due to sporting activities and contact sports [43]. Becker et al. also reflect a similar percentage, with 52.5% of shoulder dislocations occurring due to sports. Contact sports, such as basketball and American football, are associated with the highest rates of shoulder dislocations, producing 16.4% and 15.6% of all shoulder dislocations respectively; cycling comes in third with 9%. When comparing the occurrences of shoulder dislocations among males, the number of dislocations due to sports was significantly higher than those due to other causes with 86.1% and 56.7%, respectively (P=<0.01). Furthermore, sports-related shoulder dislocations were higher below the age of 20 when compared to non-sports-related dislocations, which were noted to be higher above the age of 40. Similarly, the rate of recurrent shoulder dislocations and shoulder instability was higher among patients below the age of 30, especially for those involved in high-level sports [3].

Evidently, a repetition of the benefit of external rotation immobilization among the 20-40-year-old age subgroup over various trials can be seen. Itoi et al. reported that 82% of people who were treated with external rotation immobilization returned to pre-injury sports while only 58% from the internal rotation group returned to pre-injury sports [16]. Murray et al. compared recurrence rates in overhead and contact sports and competitive sports between IR and ER groups. In the ER group, there was a 30% recurrence rate with overhead and contact sports and 21% with competitive sports, while in the IR group, there was a 53% recurrence rate with overhead and contact sports and 55% with competitive sports [12]. Although these results did not prove to be statistically significant, a trend can be seen for the better with the use of external rotation among those participating in sporting activities.

It must be kept in mind that recurrent dislocations predispose patients to higher rates of arthropathy when comparing them to patients with one-time shoulder dislocations. This is particularly important in athletes participating in high-energy sports who are prone to recurrent dislocations and events of glenohumeral instability throughout the competitive season. In order to prevent further capsulolabral damage, surgical stabilization may be recommended, especially in these groups of athletes [52]. According to Kirkley et al., as cited by Gurney-Dunlop et al., early surgical stabilization would be recommended as the treatment of choice in patients below the age of 30 and in elite athletes participating in high-energy sports and in sports that have a high rate of shoulder dislocations such as basketball and rugby [9,53]. Treatment choice among athletes, whether conservative treatment or surgical stabilization, would also depend on several other factors. If the athlete is expected to return to sport during the same season, it would be necessary to restore function quickly and efficiently but also safely. While aiming to reduce time away from the field, prevention of further injury is of utmost importance. Conservative management can be attempted in these athletes so long as they are not involved in overhead or contact sports. Given the athlete is able to return to sport-specific drills without difficulty, pain, or instability, they would be expected to return to their sport 7-21 days after completing their period of shoulder immobilization.

Should conservative management fail to prevent instability or fail to allow the athlete to perform their sport-specific drills, the athlete can then be offered in-season surgical stabilization as the next treatment option. Alternatively, surgical stabilization can also be offered earlier on if the athlete encounters recurrent instability during the season and is hasty in returning to the competitive season. Early surgical repair has shown to be clinically effective in improving patient outcomes, especially those with large bony defects and recurrent instability [9]. A person with a previous episode of a shoulder dislocation is expected to have an average of 3.5 more dislocations. And while the immediate cost of shoulder surgical stabilization may appear high, in cases where it is expected to prevent these two to three further dislocations within a specific time frame, it can minimize healthcare costs through reduced visits to the emergency department, ultimately proving to be cost-effective. To put this into better perspective, Comadoll et al. discuss the average costs of a closed surgical reduction provided at a medical facility, where a closed reduction without sedation is about $973 and could reach up to $3744 when using procedural sedation. This averages to be approximately $2207 for one visit and up to an average of $6621 for 3 recurrent episodes of shoulder dislocation. Arthroscopic repairs, such as the Bankart repair or the Latarjet procedure, which have re-dislocation rates of 10% and 5%, respectively, cost about $7800 [10]. Eighty-eight percent of athletes, on average, participating in collision (88.2%) and overhead sports (90.3%), return to play following the Latarjet procedure within 3 to 8 months; 72.6% of whom return to pre-injury levels [54]. With such low rates of recurrent dislocations and high rates of return-to-play, not only might this benefit general high-risk groups, but this might be the optimum treatment of choice in athletes who participate in collision or contact sports and are therefore considered to be at a higher risk of sustaining recurrent dislocations. Similarly, an average of 86% of athletes who underwent open or arthroscopic Bankart repairs returned to sports; 78% of which returned to pre-injury levels [55,56]. According to an article by Aboalata et al., patients who underwent arthroscopic stabilization appear to have an overall rate of recurrent instability of 18%, with rates as high as 39% in patients below the age of 20 [57]. Further long-term studies comparing conservative management outcomes to outcomes following arthroscopic or open-surgical shoulder stabilization are required to be able to personalize treatment plans according to the population. 

A summary of key findings gathered from the various studies is shown in Table 1.

**Table 1:** Summary table of pooled research IR: internal rotation, ER: external rotation, Add: adduction, Abd: abduction, SR: systematic review, SRMA: systematic review meta-analysis, MA: meta-analysis, MRI: magnetic resonance imaging, LLC: labroligamentous complex

Author(s)	Type of Study	Sample Size	Age Range (Mean) Years	Shoulder Injury	Degree IR/ER	Results	ER Brace Used	Weeks of Immobilization	Compliance Rate	Recurrence Rate IR	Recurrence Rate ER	Age Group most affected	Follow-up (Mean) Months	Follow-up Results	Bias/Quality of Evidence
Itoi et al., (1999) [25]	Cadaveric study	10			From full IR to full ER in 10° increments	In adduction, the edges of the simulated Bankart lesion were coapted in the range from full IR to 30° of ER									
Itoi et al., (2001) [17]	MRI study	19		Primary and recurrent shoulder dislocations	29° IR through to 35° ER	Immobilization in ER better approximates Bankart lesions to the glenoid neck than does IR									
Hatrick et al., (2002) [18]	Cadaveric study	10	45-60			No contact force between the glenoid and glenoid labrum in IR; contact force maximum at 45° ER									
Itoi et al., (2003) [16]	Prospective, randomized	40	17-84 (39)	Primary anterior shoulder dislocation; greater tuberosity fracture, glenoid fracture	10° ER	0% recurrence in the ER group	ER immobilization was performed using a wire-mesh splint with a sponge covering	3	77.5%; no significant difference in compliance between groups (P =0.70)	6/20(30%)	0/20(0%)	The recurrence rate is higher in the <30 age subgroup in the IR group. 0 in the ER group			
Miller et al., (2004) [24]	Cadaveric study	10			60° IR, neutral rotation, 45° ER	No detectable contact force in IR. The contact force between the labrum and glenoid increased as shoulders passed through neutral rotation; maximum contact force at 45° ER, and may influence the healing of Bankart lesions.									
Hart and Kelly (2005) [27]	Prospective, arthroscopic study	25	15-57 (24.9)	Bankart lesions, Hill-Sachs lesions, Osteochondral loose bodies, labral tears, rotator cuff tears, SLAP lesions	30°Abd-60°ER	Add-IR caused persistent displacement of Bankart lesions in 92% of patients. The best reduction was found with 30°Abd-60°ER		4							
Itoi et al., (2007) [20]	RCT	198	12-90(35)	Primary anterior shoulder dislocation; no fractures	10° ER	42% IR, 26% ER (P=0.033)	Immobilization in external rotation was performed with a wire-mesh splint covered with a sponge.	3	IR 53%, ER 72%	31/74(42%)	22/85(26%)	Relative risk reduction was 46.1% for the <30 age subgroup using ER	24; rate of follow-up 80.3%		Low risk of bias
Itoi et al., (2022) [46]	RCT based on Itoi et al., (2007)					At the 18-year follow-up, no significant difference in the recurrence rate between the ER and IR groups (P=0.100)								28% available at the 18-year follow-up	
Finestone et al., (2009) [50]	RCT	51	17-27		15°-20° ER	No difference in recurrence rate IR vs ER		4	Brace removed to shower and change clothes	10/24(41.7%) (P=0.74)	10/27(37%)		24-48 (33)		Randomization technique not mentioned. No blinding. High risk of bias
Liavaag et al., (2009) [26]	RCT, MRI	55	16-40(27)	Primary shoulder dislocations: greater tuberosity fractures, Bankart lesions present on MRI	15° ER	ER reduced the number of Bankart lesions (P=0.04) and reduced separation (P=0.03). No difference in labral displacement between groups	Don Joy Ultrasling ER, 15° version	3	16 hours/day				12; 97.9% follow-up rate		Single blinded, randomized
Siegler et al., (2010) [28]	Prospective, MRI	23	17-40(32)	Rotator cuff tears, humeral notches, glenoid lesions, hemarthrosis	ER Range 12-60°; ER Mean 37°	ER reduced hemarthrosis, anterior capsule detachment and labral lesions									
Taskoparan et al., (2010) [51]	RCT	33	15-75(32)		10° ER and Add	ER reduced the recurrence rate in the 21-30 age subgroup	Unknown splint fixated in 10° ER and Add	3	Brace removed to shower and change clothes	29.4% (5/17) (P=>0.05)	6.3% (1/16)	21-30 years (P=0.035)	6-41(20.9) months		Blinding not mentioned, randomized according to odd or even numbers; high risk of bias
Liavaag et al., (2011) [47]	RCT	188	16-40	Primary shoulder dislocations without fractures or nerve injuries	15° ER	No difference in recurrent rate IR vs ER	Don Joy Ultrasling ER, 15° version, Vista, CA	3	Brace removed to shower and change clothes, >/16 hours/day; IR 47.4%, ER 67.7% (P=<0.05)	23/93(24.7%)	28/91(30.8%)	The recurrence rate was highest in the 16-22 age subgroup	24		Low risk of bias
Heidari et al., (2014) [33]	RCT	102	15-55(35.7)		15°Abd-10°ER	33.3% in the Add-IR group and 3.9% in the Abd-ER group had recurrence (P <0.001)	Stabilizer brace using hard polyethylene, Velcro, adjustable metal bar	3	Brace removed to shower and change clothes			31-40 age subgroup had a higher rate of re-dislocation	24; interview at 33 months	No patients were lost to follow-up at 24 months	Blinded, randomized, low risk of bias
Königshausen et al., (2014) [31]	Prospective MRI-controlled study	26	15-45 (29.3)	Primary anterior shoulder dislocations without any lesions	15°- 30° ER	MRI revealed the position of the labrum relative to the glenoid rim improved significantly with ER vs IR (P=0.001 for dislocation, P=0.011 for separation of the LLC)	Don Joy Ultrasling ER, 15° version, Vista, CA	3							
Liu et al., (2014) [44]	SRMA	663	Dec-90			No significant difference was observed in the recurrence rate at all ages p=0.067		03-Apr					.(15.5)	An acceptable dropout rate (<20%) was demonstrated in all trials	Several trials included had a degree of bias due to lack of blinding
Vavken et al., 2014 [23]	SRMA	471				No support for ER over IR in all age groups		03-Apr					15.6-36		80% used randomization, 20% used blinded outcome assessment
Whelan et al., (2014) [32]	RCT	60	14-35(23)	Primary traumatic anterior shoulder dislocations without large bony lesions or polytrauma; Hill-Sachs lesions and small bony Bankart lesions were included	0°Abd, 0-5° ER/ 0°Abd, 70-80° IR	No difference in the rate of recurrent instability between groups. A trend toward the benefit of ER after a primary dislocation in older patient cohorts is suggested (P =0.06)	DonJoy; DJO, LLC, Vista, CA	4	The brace removed for showering and physiotherapy; 80% overall compliance	8/25(32%)	6/27(22%)		12-43(25)	17% loss of follow-up	Low risk of bias, single-blinded, randomized
Itoi et al., (2015) [29]	Controlled laboratory study, MRI study	37		Primary shoulder dislocations	Add-IR, Add-30°ER, 30°Abd-30°ER, 30°Abd-60°ER	Abd-ER demonstrates improved reduction of the Bankart lesions									
Momenzadeh et al., (2015) [30]	Prospective, MRI study	20	<40 (26.4)	Primary anterior shoulder dislocation without fractures	10° ER	Statistically significant reduction in separation in the ER group (P=0.028)	Shoulder spica cast with 10° ER	3					3 weeks	25% loss of follow-up	Randomization for grouping; blinding during MRI reporting
Whelan et al., (2016) [45]	SRMA	632	20.3-35.9(30)			No difference in recurrent rate IR vs ER. Overall recurrence rate 29.1% (169/581)		03-Apr	No significant difference in compliance ER vs IR (P=0.43); ER 218/287 (76%), IR 180/273 (66%)	96/284 (33.8%) (P=0.15)	73/297 (24.6%)	No significant difference with age subgroup analyses	24-48		
Braun and McRobert (2019) [58]	SRMA	704	12-90 (29)			Insufficient evidence to draw a conclusion on ER vs IR		03-Apr		73/243 (30%)	55/245 (22%)		≥ 12 (24)		All trials included had a degree of bias due to lack of blinding
Murray et al., (2020) [12]	RCT, MRI	47	18-47(27)	Primary anterior shoulder dislocations, Bankart and other lesions found on MRI	0°Abd-15°ER	The recurrence rate did not show a statistically significant difference overall but showed a significant reduction in the 20-40-year subgroup. Recurrence rate reduced with healed labrum regardless of group (p = 0.001).	Don Joy Ultra Sling II ER, DJO Global, Vista, CA	3	About 90% compliance in both groups	Overall 47.8%; 20-40 age group 50%	overall 29.2%; 20-40 age group 6.4%	Significant difference favouring ER in the 20–40 age subgroup (p = 0.044). Patients <20 showed the highest recurrence rate; no recurrence occurred within 2 years in patients >40	24	The follow-up rate in the IR and ER groups was 92% and 96%, respectively	Low risk of bias; low power; recall bias for compliance rating
Shinagawa et al., (2020) [21]	SRMA	817	Mean age range 20.3-37.5			ER significantly reduced the recurrence rate compared to IR (P =0.007).		3-4.5	Ranging from > 16 hours/day to removing only for showers, 2 unknown compliance. Excluding low compliance studies revealed a lower recurrence rate in the ER group (P =0.01)	130/373(34.9%); studies with > 24 months follow-up had an IR recurrence rate of 40.1% (69/172)	84/390(21.5%); studies with > 24 months follow-up had an IR recurrence rate of 21.9% (41/187)	ER is significantly more effective than IR in the 20-40 age subgroup but not in the <20 age subgroup	≥ 6 months		The methodological quality of the trials ranged from low to moderate
Zhang et al., (2020) [38]	SRMA	1042				ER reduces the recurrence rate and prevents complications (P=0.004)		03-Apr	No significant difference in compliance between ER and IR			ER significantly reduces recurrences in the <40 age subgroup; no difference for the>40 age subgroup	<1-48		All trials were generally assessed low in quality and contained the risk of bias
Hurley et al., (2021) [19]	MA	795	.(29)			Recurrence rate lower in ER (22.2% vs 33.4%) (P=0.02)		03-Apr				The recurrence rate in the 20–40 age subgroup was significantly lower in the ER group than the IR group (12.1% vs 31.4%) (P=0.006)	(25.5) months		5 studies had a low risk of bias, 4 studies had a high risk of bias. All had a variation of randomization but not all were blinded

Limitations

Studies of different levels of evidence and quality were used to gather an abundance of currently available data, and therefore the level of bias of each study was unavoidably included. Itoi et al. advocate for sooner immobilization for optimum results but only a few studies have outlined the accepted time to immobilization in their inclusion criteria [20]. This review did not take into consideration the time from shoulder reduction to ER immobilization. Furthermore, the time to re-dislocation after the period of ER immobilization was also not considered but is of importance. Data analysis and comparing immobilization to surgical stabilization were not covered among the objectives of this literature review but would be beneficial to delve into to propose optimum treatment plans, especially for those athletes eager to return to sport. Patient-reported outcome measures will aid in identifying the possible success rates of ER immobilization; this was omitted from the review due to time restrictions.

While this review included a greater number of level I and II research, the cadaveric and imaging studies analyzed and included in this review are considered to be laboratory-controlled studies and therefore fall under a level of evidence of V. The SRMA by Vavken et al., which did not support external rotation immobilization in their conclusion, had focused mainly on level II evidence [23]. On the other hand, Shinagawa et al. included level II and III evidence within their SRMA and results favored ER immobilization [21]. In the latest SRMA by Hurley et al., which also supported ER immobilization, four of the included research articles were categorized as having a high risk of bias while five of them were categorized as low risk [19]. This was similarly recorded in the Cochrane review by Braun and McRobert, whereby they considered all the articles in their review as very low levels of evidence [58]. Despite papers being considered level I or II evidence for carrying out a systematic review and meta-analysis, if the data pooled also includes lower levels of evidence, this will ultimately affect the concluded outcome. Of note, the level I evidence RCT produced by Liavaag et al. supported Itoi and colleagues’ findings of external rotation immobilization, reducing the number of Bankart lesions. However, at this time, a definitive conclusion favoring external rotation immobilization over internal rotation remains difficult to draw.

## Conclusions

The current literature presents sufficient evidence to support anatomical reduction and coaptation of structures of the shoulder when immobilization is in external rotation. However, there remains insufficient evidence to universally apply this in clinical subjects. Cadaveric and imaging studies support greater degrees of external rotation with or without abduction to achieve better contact force of the underlying structures. In clinical subjects, the degree of external rotation has yet to be uniformly implemented, maintained, and extensively evaluated. Further large high-quality trials are needed to study the ideal Abd-ER immobilization degree that will aid in reducing the recurrence rates of shoulder dislocations. More studies on various braces and their ability to maintain the intended degree and position of immobilization, to provide comfort, and to be reapplied with ease are also essential. After an injury, granulation tissue is typically formed between days 7 to 10 and increases its tensile strength by the third week. While some researchers report no difference in the recurrence of shoulder dislocations when comparing one week and three weeks of immobilization, the majority of clinical trials have leaned toward using three weeks as the period of immobilization. There is no clear evidence to support immobilization for longer than three weeks. Furthermore, the potential benefits of early surgical stabilization in athletes, when compared to conservative shoulder immobilization, require further research.

People below the age of 40 are at a high risk of recurrent dislocations. The male gender appears to be an additional risk factor. Although most shoulder dislocations may be related to sports activity or contact sports in particular, sports, in general, has failed to show an impact on the rate of recurrent shoulder dislocations. Further studies delving into the risk factors and confounders are needed. Clinical trials have not been able to consistently report a reduction in recurrence rates using external rotation immobilization. External rotation immobilization may benefit a specific population, particularly those that fall in the 20-40 year age group, with a specific injury pattern, perhaps the primary anterior shoulder dislocations that are typically associated with Bankart lesions, greater tuberosity fractures, or hemarthrosis, and hence further studies are required to determine who will benefit the most from such intervention. Currently available research presents discrepancies among the variables used. In order to identify possible beneficial outcomes of external rotation immobilization, it is advisable that these variables be isolated or standardized in future studies to allow the focus to be solely on the method of immobilization.
